# Taking ACTION to reduce pain: ACTION study rationale, design and protocol of a randomized trial of a proactive telephone-based coaching intervention for chronic musculoskeletal pain among African Americans

**DOI:** 10.1186/s12891-016-1363-6

**Published:** 2017-01-13

**Authors:** Rozina H. Bhimani, Lee J. S. Cross, Brent C. Taylor, Laura A. Meis, Steven S. Fu, Kelli D. Allen, Sarah L. Krein, Tam Do, Robert D. Kerns, Diana J. Burgess

**Affiliations:** 1 School of Nursing, AGH Cooperative, University of Minnesota, Minneapolis, MN USA; 2 Center for Chronic Disease Outcomes Research (a VA HSR&D Center of Excellence), Veterans Affairs Medical Center, Minneapolis, MN USA; 3 Department of Medicine, University of Minnesota, Minneapolis, MN USA; 4 Center for Health Services Research in Primary Care, Veterans Affairs (a VA HSR&D Center of Excellence), Veterans Affairs Medical Center, Durham, NC USA; 5 Department of Medicine & Thurston Arthritis Research Center, University of North Carolina at Chapel Hill, Chapel Hill, NC USA; 6 Center for Clinical Management Research, Veterans Affairs Healthcare System, Ann Arbor, MI USA; 7 Department of Internal Medicine, University of Michigan, Ann Arbor, MI USA; 8 Departments of Psychiatry, Neurology and Psychology, Yale University, New Haven, CT USA; 9 Pain Research, Informatics, Multimorbidities and Education (PRIME) Center of Innovation, VA Connecticut Healthcare System, West Haven, CT USA

**Keywords:** Chronic musculoskeletal pain, African American, Veterans administration, Randomized control trial

## Abstract

**Background:**

Rates of chronic pain are rising sharply in the United States and worldwide. Presently, there is evidence of racial disparities in pain treatment and treatment outcomes in the United States but few interventions designed to address these disparities. There is growing consensus that chronic musculoskeletal pain is best addressed by a biopsychosocial approach that acknowledges the role of psychological and environmental factors, some of which differ by race.

**Methods/Design:**

The primary aim of this randomized controlled trial is to test the effectiveness of a non-pharmacological, self-regulatory intervention, administered proactively by telephone, at improving pain outcomes and increasing walking among African American patients with hip, back and knee pain. Participants assigned to the intervention will receive a telephone counselor delivered pedometer-mediated walking intervention that incorporates action planning and motivational interviewing. The intervention will consist of 6 telephone counseling sessions over an 8–10 week period. Participants randomly assigned to Usual Care will receive an informational brochure and a pedometer. The primary outcome is chronic pain-related physical functioning, assessed at 6 months, by the revised Roland and Morris Disability Questionnaire, a measure recommended by the Initiative on Methods, Measurement, and Pain Assessment in Clinical Trials (IMMPACT). We will also examine whether the intervention improves other IMMPACT-recommended domains (pain intensity, emotional functioning, and ratings of overall improvement). Secondary objectives include examining whether the intervention reduces health care service utilization and use of opioid analgesics and whether key contributors to racial/ethnic disparities targeted by the intervention mediate improvement in chronic pain outcomes Measures will be assessed by mail and phone surveys at baseline, three months, and six months. Data analysis of primary aims will follow intent-to-treat methodology.

**Discussion:**

We will tailor our intervention to address key contributors to racial pain disparities and examine the effects of the intervention on important pain treatment outcomes for African Americans with chronic musculoskeletal pain.

**Trial registration:**

ClinicalTrials.gov: NCT01983228. Registered 6 November 2013.

## Background

Approximately 100 million adults in the United States (US) suffer from chronic pain, and musculoskeletal pain is the most common type of chronic pain [1]. Moreover, rates of chronic pain has been rising in the US, and are expected to continue to rise [1]. This is particularly worrisome because chronic pain is associated with poorer self-reported health status, worse mental health, lower levels of employment, and higher use of medical services [1]. Indeed, the Institute of Medicine estimates the annual cost of chronic pain to be $560 to $635 billion, due to direct cost of medical care, lost productivity, costs associated with disability programs and the burden chronic pain places on families [1].

Presently, there is evidence of racial and ethnic disparities in pain in the United States but few interventions designed to address these disparities [1–3]. We consider racial/ethnic disparities in *pain* to be a type of health disparity, defined as a difference in health status that systematically and negatively impacts racial/ethnic minority groups [4]. We define racial disparities in *pain treatment*, using the definition of health care disparities from the Institute of Medicine report, *Unequal Treatment*, as “differences in the quality of health care that are not due to access-related factors or clinical needs, preferences or appropriateness of intervention” [5]. Importantly, contributors to racial disparities in pain are complex and multi-level, including but not limited to racial disparities in pain treatment. We focus on African American/white disparities in pain, although there are also differences found for other racial/ethnic minority groups [1].

A number of contributors to racial disparities in pain occur at the level of the *healthcare system*. African American patients in the United States are more likely to experience barriers to accessing and *utilizing healthcare*. African American patients are more likely than whites to have unmet medical needs due to myriad factors, including lack of insurance and underinsurance [6], experiences of discrimination within and outside the healthcare system (associated with avoiding and delaying care), poorly coordinated care, lack of a primary care provider, and logistical barriers (e.g., lack of childcare and transportation) [6, 7]. African American patients also experience *racial disparities in pain treatment*. They are more likely than whites to have their pain discounted and underestimated, are less likely to be screened for pain, and more likely to be undertreated and to receive less or no analgesia [1, 8–10]. African American patients also are more likely to experience *poor quality communication with their providers*, which adversely affects the quality of pain treatment [1, 11].

*Environmental factors* may also contribute to racial disparities in pain, through multiple pathways. There is growing evidence that experiences of racial discrimination, experienced within and outside healthcare, are associated with greater pain, although the mechanisms by which this occurs are not fully understood [12–18]. African Americans are more likely to experience barriers that impede effective self-management, such as exercise. For example, in the United States, African Americans are more likely to reside in neighborhoods low in “walkability” [19, 20].

*Psychological factors* may also contribute to disparities in pain, by reducing the use of effective self-management strategies [8–10]. This includes patient beliefs and attitudes that contribute to poor pain outcomes (e.g., pain-related fear of movement, low perceived control over pain, lower self-efficacy in coping with pain), which African Americans are more likely to hold [1, 21, 22].

There is growing consensus that chronic musculoskeletal pain is best addressed by a biopsychosocial approach that acknowledges the role of psychological and environmental contributors to pain [23–28], some of which differ for African Americans and hence contribute to disparities. Our goal is to test an intervention to improve pain outcomes among African American patients. This intervention could be targeted to African American patients (e.g., to healthcare systems that predominantly serve African American patients), as a way of reducing disparities. However, the intervention itself is not designed to test whether it reduces disparities since we also expect the intervention to benefit non-African Americans.

### Conceptual framework

#### Rationale for the intervention

The intervention was based on several lines of research evidence. First, physical activity can reduce chronic musculoskeletal pain and improve function [16–18]. Second, proactive telephone outreach (in which a counselor reaches out to patients to offer them the intervention, rather than requiring the patients to seek out care) can address environmental barriers that lead to lower levels of utilization of care among African Americans [19]. Third, pedometer-based walking programs are effective at increasing walking for various groups [20, 21], including African Americans [22–25]. Fourth, making an action plan (specifying when, where, and how the behavior will be performed) increases the likelihood that individuals will perform intended behaviors and overcome psychological and environmental barriers [24, 25]. Fifth, motivational interviewing may be an effective intervention strategies for improving pain self-management and reducing pain, by intervening on psychological contributors, which are more prevalent among African American patients experiencing pain [26]. Finally, there is evidence that African American patients desire non-pharmacological approaches to pain treatment, including exercise [27].

**Fig. 1:**
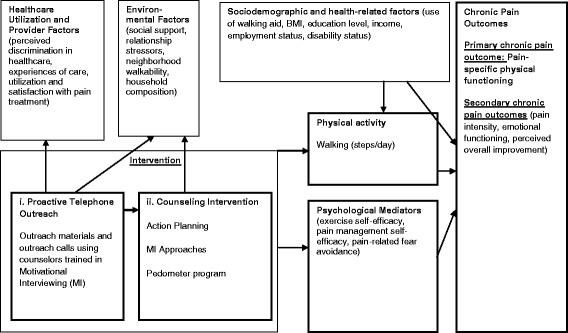
Conceptual Model

Given the psychological and environmental contributors to racial disparities in chronic pain treatment, we developed an intervention that addresses the multiple contributors to chronic pain that disproportionately affect African American patients (see Fig. 1 for a depiction of our hypothesized contributors to racial disparities in pain). The intervention is based on a biopsychosocial model and has several components. Action planning and MI approaches are used to overcome psychological barriers to exercise (low self-efficacy for exercise and coping with pain, pain-related fear) and promote change [28–31]. Self-efficacy is particularly important to include since a recent review identified self-efficacy as the strongest predictor of intentions to walk more [28]. Pedometers are used as a tool to promote walking through feedback, goal setting, and monitoring [20, 21]. Counselors will use a variety of methods to improve coping skills (i.e., problem-solving, counseling, planning to overcome barriers) [3], facilitate supportive planning (i.e., making plans to increase and strengthen helpful factors) [32], and shared planning that involve friends and family members [19]. Although the proposed intervention was designed to address contributors to pain that African American patients are especially likely to experience, we also expected expect that non African American patients in our veteran populations will also experience these contributors, albeit to a lesser extent. For example, non-African American patients with musculoskeletal pain also experience frustration with their treatment of pain within the current healthcare system and report feeling stigmatized by their providers [1]. Veterans are also considered to be a vulnerable group that is at greater risk for pain and poor pain treatment compared to non-Veterans [1]. Hence, while this intervention may be more effective for African American patients, it should also benefit non-African American patients in the Veterans Affairs Healthcare System. For this reason, we believed it was valuable to test this intervention on non-African American Veterans. Although we do not have statistical power to test whether this intervention reduces racial disparities in pain, we planned to explore whether the intervention appears to reduce racial disparities in pain outcomes. If this intervention is effective, we can examine whether it reduces disparities in a future study that would be powered to test this as a primary outcome, using data from this study to inform our sample size calculations.

### Key intervention components

#### Intervention condition

##### Mailed recruitment materials

Participants assigned to the intervention group will receive personalized engagement materials, including a letter and brochure describing the program and the benefits of walking to help manage pain. Materials include targeted messages to enhance persuasive appeal among this population and were developed using 4 focus groups among African American patients at the Atlanta VA Medical Center (VAMC). This approach to developing engagement materials was successfully used by Fu and colleagues in a prior study with Veterans [25].

##### Telephone sessions

Intervention participants will be asked to complete six telephone counseling sessions over a 10 week period (with a 12 week absolute cut off time point) using a patient workbook (adapted from French et al.) [15, 16] with visual aids and worksheets they complete during the counseling sessions. For example, one visual aid is a figure that describes a cycle of pain leading to inactivity which may contribute to deconditioning, and in turn, can contribute to more pain when being active. An example of a worksheet is a table divided by day in which to write number of steps, details of walks, time spent walking, and successes and/or challenges. Participants are expected to receive approximately 180 min of total counselor time during the study.

The action planning component of this intervention was based on a protocol and structured curriculum developed by French and colleagues. This program was found to be effective at increasing walking in several trials in a non-clinical population (healthy volunteers) [28–30]. It also incorporates techniques developed in an intervention that used action planning to promote physical activity among patients with chronic low back pain [18]. Participants were coached to create and write action plans for their proposed walking activity, during the week(s) between counseling sessions, using a template contained in their workbooks (which prompted them to indicate when, where, and with whom they would walk). In order to adapt this component to our target population, counselors are trained to coach patients in developing plans to overcome common environmental barriers to walking experienced by members of racial minority groups (e.g., neighborhoods that lack walking paths, poor access to parks, lack of time) [4, 24, 25, 33]. We specifically address fear of movement and promote self-efficacy for walking during the action planning process by incorporating these topics and MI approaches into the action planning portion of the sessions.

The pedometer component of this intervention was based on the work of French and colleagues [16], and Krein and colleagues [21], which use modest goals to assist participants with gradually increasing their step counts [16, 21]. For the duration of the counseling period, intervention participants will be asked to wear their pedometer and maintain a weekly pedometer diary, which is a protocol demonstrated to be feasible in the “Fit for Life” [22] and H.U.B. City Steps studies [29]. The structure and intensity of our intervention is based on prior interventions that successfully increased walking and improved pain outcomes [14, 15, 30, 34]. We particularly chose to balance practical concerns about future dissemination, while accommodating our intervention components and providing a sufficient dose of counselor time.

##### Counselor training

Counselors have at least a Master’s degree in a counseling-related field (e.g., counseling psychology, clinical social work) and receive at least 15 h of training by an expert to prepare them to deliver the intervention using a structured training manual. Training involves reviewing the treatment manual, role playing, attending a Motivational Interviewing course and review of audio-taped sessions with feedback. Session tapes are reviewed to assess fidelity to the protocol and to provide feedback to counselors. Counselors will participate in case consultation throughout the course of the intervention to prevent “drift” from the manualized treatment. An expert in counselor training will meet with the study counselors about every week to provide corrective feedback and ongoing support. In order to ensure treatment integrity, all treatment sessions will be audiotaped and 10% will be randomly selected and reviewed by trained coders to assess both the use of treatment concordant strategies and the absence of commonly used treatment strategies not included in the manualized treatment.

#### Usual care control condition

Participants randomized to the Usual Care (UC) control condition will receive pedometers and an informational brochure about the benefits of walking. They will be instructed (via a mailed postcard and reminder phone calls) to wear the pedometer and to record their pedometer readings on logs over 7 days at the baseline assessment, three months and six months follow-up time points. They will report these step counts during the surveys, a procedure which has been used successfully in previous studies with similar populations [8] (Burgess 2012, unpublished data). Although the pedometer is an enhancement beyond what is received in Usual Care, we decided to provide pedometers to participants assigned to the control condition in order to determine whether the intervention increases walking (as self-reported measures have not been shown to be reliable). In addition, past studies suggest that pedometers in isolation are unlikely to result in a sustained increase in walking among generally sedentary individuals [20], and the use of pedometer self-monitoring in research does not lead to increased physical activity [24].

### Prior studies that address acceptability, feasibility, and potency of key components of intervention

Our research team has conducted several studies that address acceptability, feasibility, and potency of key components of intervention. The “Self-Management of OsteoArthritis in Veterans” (SeMOA) study [35] was a self-management intervention that utilized Proactive Telephone Outreach and Telephone-Based Self-Management to improve pain outcomes for non-white (mostly black) and white VA patients with hip and knee OA. This population of black VA patients with OA is similar to our target population (black VA patients with MSK pain). The intervention consisted of 12 monthly phone calls by a health educator, who reviewed education materials and provided support for developing individualized goals and Action Plans related to OA management. Results of SeMOA demonstrated the feasibility of conducting a telephone-based self-management trial to reduce pain among black Veterans, using a proactive recruitment strategy based on identifying patients from the electronic medical record, and following up with a telephone screener. The study met its recruitment goals and completion rates among non-white (mostly black) OA Veteran patients were high (89%) and comparable to rates among whites (91%). Process evaluation of SeMOA demonstrated that black Veterans perceived the intervention to be helpful for improving their OA symptoms and were more likely than whites to view it as helpful [8]. The SeMOA intervention led to a clinically relevant improvement in pain among black Veterans with OA; at 12 months, the mean Arthritis Impact Measurement Scales-2 (AIMS-2) pain score (measuring pain, affect, and physical function) in the osteoarthritis self-management group was 0.4 point lower (*P =* 0.105) than in the UC group and 0.6 point lower (*P =* 0.007) than in the health education (HE) group. The mean visual analog scale pain score in the intervention group was 1.1 points lower (*P <* 0.001) than in the UC group and 1.0 point lower (CI, −1.5 to −0.5 point; *P <* 0.001) than in the HE group [9, 10].

The “Tailoring Coping Skills Training (CST) for African Americans with Osteoarthritis” pilot study involved focus groups and a pilot trial to 1) assess the cultural appropriateness of an intervention using specific CBT techniques to improve coping skills and improve pain outcomes among black veterans with OA, 2) tailor the intervention and modify it for telephone, and 3) perform a pilot trial of the intervention. These CBT techniques are part of the proposed intervention (e.g., cognitive restructuring, relaxation, imagery, activity pacing). Focus groups showed that black Veterans viewed the intervention as culturally appropriate. Tailoring involved lowering literacy levels of patient materials. The pilot trial of CST demonstrated individual improvements on the AIMS-2 (among a racially mixed sample of Veterans with OA).

Pedometer-Mediated Walking program to reduce pain among chronic musculoskeletal pain patients. In the “Veterans Walk to Beat Back Pain” study, 229 Veterans with back pain were randomly assigned to a pedometer-mediated walking intervention, administered over the internet, or Usual Care (which included general back pain education and a pedometer). Potentially eligible participants were identified both through provider referrals and by using VA electronic medical record data. Findings from this study showed that > 90% of patients in both groups completed 6-month assessments, with intervention patients reporting significantly less back pain-related disability compared to controls (Roland Morris Disability Scores: 7.2 vs. 9.2, *P =* 0.01) as well as lower pain scores (4.7 vs. 5.2, *P =* 0.06), and greater average step counts [36]. This project demonstrates the feasibility and acceptability of conducting a trial of a pedometer-mediated intervention for chronic pain patients, and speaks to the potency of this intervention in reducing pain related disability as well as pain levels. However, this study was conducted on a primarily non-minority population (who were required to have a home computer with internet access), and was not designed to address barriers faced by minority VA patients, which the present study is designed to address.

Cognitive Behavioral Therapy (CBT) techniques and exercise for chronic MSK pain. Dr. Heapy and Dr. Robert Kerns have reported a number of studies that demonstrate the feasibility and acceptability of conducting CBT trials with Veterans with chronic pain (including the use of CBT to promote physical activity), as well as formative research that provided information about the types of coping skills most preferred by patients with chronic pain (which included exercise). These studies include: 1) an evaluation of the effect of a tailored cognitive-behavioral approach to the management of chronic back pain on promoting adherence to therapist recommendations for pain coping skill practice; 2) a randomized controlled trial of cognitive-behavior therapy for painful diabetic peripheral neuropathic pain; and 3) a study testing the efficacy of interactive voice response, for delivering CBT for chronic low back pain [37–40].

## Methods/Design

The goal of this randomized controlled trial is to investigate the effectiveness of the intervention in improving the pain-related function of African American patients with chronic musculoskeletal pain through a proactive coaching program designed to increase walking. Data collection will occur at baseline (randomization) and at three and six months post-randomization.

The specific study aims are:

Primary Aims: To test the hypothesis that compared to UC, a proactively delivered walking intervention targeted to African American VA patients with chronic musculoskeletal pain will:

1. improve pain-related physical functioning (primary outcome),
2. improve emotional functioning, pain intensity, and ratings of overall improvement (first secondary outcome)
3. increase walking as measured by step counts (second secondary outcome).

Secondary Aims are to:

1. investigate whether key contributors to racial disparities targeted by the intervention mediate improvement in chronic pain outcomes and increases in walking,
2. test the effectiveness of the intervention on non-African American patients and explore whether effectiveness differs between African American and non-African American patients, and
3. examine whether the intervention reduces the use of opioid analgesics.

This study is approved by the Institutional Review Boards (IRBs) at the Minneapolis VAMC and VA Central Office IRB in Washington, DC. ACTION is also reviewed and approved annually by the granting institution, the VA Health Services Research & Development (HSR&D) Service.

### Eligibility

#### Sample and eligibility criteria

Study participants will be recruited from the Atlanta VAMC, which we chose because it has a high percentage of African American patients. The main facility and satellite clinics have about 9,294 patients who meet our initial eligibility criteria. These criteria include having 1 hip, back or knee diagnosis code in the past year, a second hip, back or knee diagnosis code 18 months from the first one, and the Atlanta VA is identified as their preferred care location. From this cohort, we will randomly select patients (African Americans and non-African Americans) for recruitment. We will include non-African Americans in our sample for comparison purpose. Administrative race data will be verified on the brief screening survey.

Recruitment will be conducted via an introductory letter, which describes the study for potential participants and provides an option to opt out if they are not interested in participating in the study. Further, qualifying criteria will be determined through the administration of a telephone screening questionnaire. Eligible patients must have pain duration of ≥ 6 months, moderate-severe pain severity and interference with function (defined as a brief pain intensity and interference (PEG) score of ≥ 5), self-reported ability to walk at least 1 block, and be able to communicate effectively by telephone. The PEG (Pain intensity, Enjoyment of life, General activity) is an ultra-brief (three items) assessment tool for pain, which is valid, reliable and responsive to change [41–44]. The PEG is calculated using an average of the three items, each of which is measured on scale of 0–10. Scores of ≥ 5 indicates moderate to severe pain. We will not exclude patients who are prescribed medication or receiving other treatments for chronic pain. Patients who meet any of the following exclusion criteria that may interfere with the outcome assessment are ineligible: a) moderately severe cognitive impairment defined as ≥ 2 errors on a brief cognitive screener (the six-item Callahan screener that identifies cognitive impairment for potential research subjects) [12, 38] or b) anticipated back, knee, hip or other major surgery within the next 12 months. Because this study is considered minimal risk, we received a waiver of signed, written consent from the IRB. In lieu of a written consent form, participants receive an information sheet that will be reviewed orally over the phone by a research assistant and oral consent to participate is required. Eligible respondents who consent will be mailed a pedometer and a baseline survey to complete and mail back. When completed baseline surveys are received, participants will be randomized to the UC or intervention condition.

Recruitment and telephone counseling will be conducted by staff at the Center for Chronic Disease Outcomes Research (CCDOR) at the Minneapolis VAMC. Telephone counseling will be conducted separately from recruitment by separate staff.

#### Randomization

**Fig. 2:**
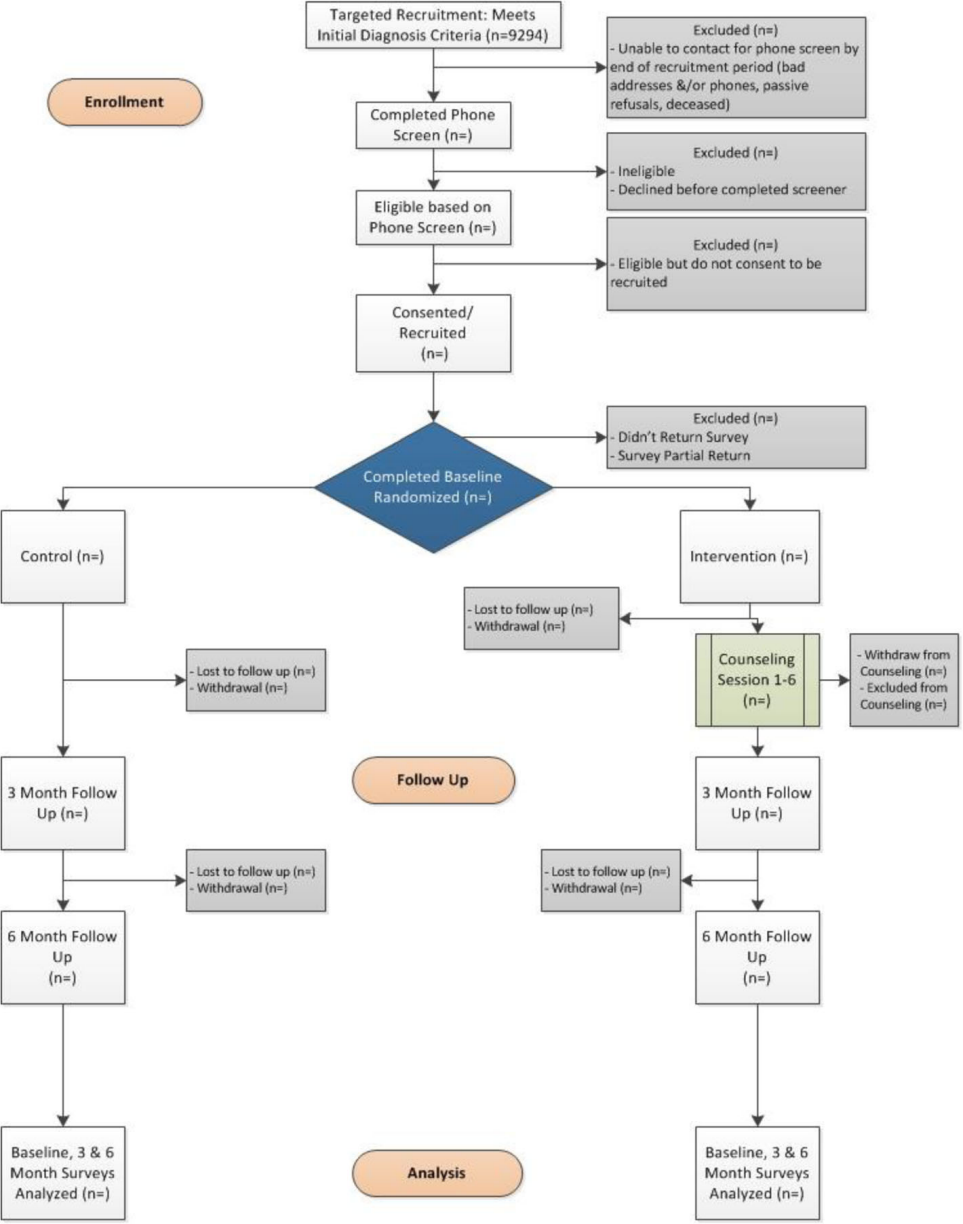
Study recruitment, enrollment, intervention, assessment, and analysis flow diagram

The primary analyses will use the sample of African American patients, and the study was intentionally designed to be adequately powered to detect effects in this core group. Additional analyses will be conducted in the non-African American sample and the overall sample. Because our primary aims focus on the sample of African American patients, the methods outlined ensure the study is sufficiently powered for this sample (see Fig. 2).

Based on our previous experience conducting interventions with African American veteran patients with chronic pain [13], we anticipate that we will be able to contact greater than 50% African American patients and that 60% will be eligible for and consent to participate in our baseline phone survey. We will recruit a total of 500 participants in this study with a minimum of 250 African American participants (about 125 in intervention group and about 125 in UC group) to ensure adequate power for the study. Among those randomized, we estimate (based on previous research conducted [12]) that 10–15% will be missing complete follow-up data. However, if the response rate is lower than expected, we will increase the necessary number of patients to enroll each month in order to accomplish our goals for available outcome data.

Participants will be randomized to either study intervention or UC (1:1) at the time that the baseline surveys are listed as complete in the study tracking application. At receipt of a completed baseline, participants will be randomized and sent letters describing whether they are in the intervention or UC group. The randomization list will be concealed from the research team within the tracking application, so team members will not know the next study assignment. In order to ensure that treatment is equally balanced on race the randomization is stratified on participant self-identified African American or non-African American status and balance in assignment over time is achieved using permuted block randomization with block sizes of 4, 6 or 8.

### Screening and measures

#### Description of measures and data collection procedures

Data collection will be conducted by mail at baseline, and a combination of mail and telephone at three and six months post-randomization. At pre-screen, potential participants will receive a pre-notice letter. About a week later, research staff will call to assess patients’ interest, answer questions, conduct an eligibility screener, go over the information sheet (in place of an informed consent form), and obtain oral consent. Participants will then be mailed a baseline survey and pedometer.

Fourteen days prior to the three and six month follow-up dates, all participants will be mailed a postcard reminding them to wear their pedometers in preparation for filling in the 7 day walking journal on the upcoming survey. A week prior to the three and six month dates, follow-up surveys will be mailed to all participants. Over the course of two weeks after surveys are mailed, non-responders will be called to ensure receipt of the survey, answer questions, and do the survey over the phone (instead of the mailed version) if the participant is willing. After 3 completed reminder calls, non-responders will be mailed another postcard reminding them to return the paper survey. In addition, at 6 months non-responders will receive a second copy of the survey in the mail along with an especially engaging cover letter. (See Tables 1 and 2 for survey measures.)

**Table 1:** Intervention targets selective contributors to racial disparities in pain

Barriers	Element of Intervention that targets barrier
Healthcare system barriers associated with access and utilization	Addressed by proactive telephone outreach
Barriers associated with provider communication	MI approaches used by counselors
Environmental barriers to exercise	Action planning and MI approaches; Coping, Supportive/facilitative, and collaborative planning
Psychological Barriers (including self-efficacy)	Action planning and MI

**Table 2:** Outcomes measures

Construct	Measure	0 mo	3 mo	6 mo
Baseline screening questions				
Race/ethnicity	Standard measures of race and ethnicity	X		
Pain intensity/interference	Brief pain intensity and interference scale (PEG)	X		
Ability to walk a block	Single-item screening question	X		
Cognitive screener	Callahan Measure	X		
Anticipated back, knee or hip or other major surgery in next 12 mo.	Single-item question	X		
Primary Chronic Pain Outcome				
Disease-specific functioning	Revised Roland and Morris Disability Questionnaire (RMD)	X	X	X
Secondary Chronic Pain Outcomes				
Pain intensity/interference	Brief pain intensity and interference scale (PEG)	X	X	X
Emotional functioning	Personal Health Questionnaire Depression Scale (PHQ-8)	X	X	X
Emotional functioning	Generalized Anxiety Disorder 7 item (GAD-7)	X	X	X
Overall improvement	Patient Global Impression of Change scale		X	X
Walking (mediator)				
Average daily total steps	Pedometer data recorded over past 7 days on patient logs	X	X	X
Utilization of pain treatment				
Use of opioid analgesics	Prescription records from Electronic Medical Records	X	X	X
Use of opioid analgesics	Survey items	X	X	X
Perceived satisfaction with pain care	Single item assessing how patients rate their quality of pain care in VA over past 6 months		X	X
Psychological factors (mediators)				
Exercise self-efficacy	Exercise Regularly Scale	X	X	X
Pain management self-efficacy	Pain Self-Efficacy Questionnaire-8 item version (PSEQ-8).	X	X	X
Pain-related fear avoidance	Fear-Avoidance Beliefs Questionnaire (FABQ) Scale 1: Fear-avoidance beliefs about physical activity	X	X	X
Environmental factors				
Social support	Social Support for Exercise: Marcus Social Support Questions	X		
Relationship Stress	Life Stressors Inventory (LISRES-A)	X		
Neighborhood walkability	Neighborhood Environment Walkability Scale (NEWS)	X		
Healthcare Utilization and Provider Factors				
Experiences of discrimination	Perceived Discrimination in Healthcare	X		
Mistrust of medical care	Evaluation of VA Care scale	X		
Sociodemographic and health-related factors				
Sociodemographic factors	Standard measures of education, income, employment status, disability status	X		
Health-related factors	BMI, use of walking aid, claim status,	X		

##### Primary and secondary chronic pain outcomes

We will assess the following core chronic pain outcome domains and measures recommended by IMMPACT: [45] 1) *The primary outcome of a pain-specific measure of functioning* will be assessed at six months using the revised version of the *Roland and Morris Disability Questionnaire (RMD)*, which is widely used in studies of chronic musculoskeletal pain and included in the IMMPACT recommendations. The RMD at three months will also be assessed as a secondary outcome. Although the original version of the RMD focused on low back pain [46], the revised version has been adapted for musculoskeletal pain more broadly, and has been validated with musculoskeletal patients [47]. The scale has good internal consistency, discriminative validity and is sensitive to change [48]. 2) Emotional functioning will be assessed by the Personal Health Questionnaire Depression Scale (PHQ-8), without the suicidality item (7 items) [49] and the Generalized Anxiety Disorder 7-item (GAD-7) scale [50]. 3) Pain intensity will be assessed by the 3-item PEG [41, 42]. 4) Participant rating of overall improvement will be assessed at three and six months by the Patient Global Impression of Change scale, a single item measure assessing patients’ views of improvement/worsening of their pain [4].

##### Walking

Walking will be measured as the number of average daily steps using pedometer readings recorded in walking logs at three and six months, based on seven consecutive days of data. We are using the Omron HJ-321 pedometer, which can be worn in a pocket, around the neck, or on a belt clip (orientation does not matter) and has been shown to be highly accurate, including in obese populations [33]. Patients will be provided with written instructions on how to use the pedometers. The instructions were assessed as part of the Atlanta focus groups and revised to be better understood by our participant population. Participants in the intervention arm will have additional support for ongoing pedometer use as part of the intervention, but the walking outcome assessment protocol is identical in both arms.

##### Psychological factors (mediators)

Pain-related fear avoidance will be measured by the fear-avoidance beliefs about physical activity subscale (Scale 1) of the Fear-Avoidance Beliefs Questionnaire (FABQ) [51]. Self-efficacy for exercise will be measured using the Exercise Regularly Scale, which includes questions asking respondents how confident they are they can do aerobic exercise such as walking three to four times each week, and how confident they are that they can exercise without making symptoms worse. Pain management self-efficacy will be measured using the eight item Pain Self-Efficacy Questionnaire (PSEQ-8) [36], which has been used in numerous studies with chronic pain patients.

##### Environmental factors

Social support for exercise will be assessed using the Marcus Social Support questions [52], and relationship stressors by the Life Stressors Inventory (LISRES-A) [53]. We will assess neighborhood walkability using the Neighborhood Environment Walkability Scale (NEWS) [54]. As part of the general demographic questions at baseline, we will also ask a question to assess household composition.

##### Healthcare utilization and provider factors

These factors will include perceived discrimination in healthcare (associated with delay of healthcare and unmet medical needs), which will be assessed using the Perceived Discrimination in Healthcare Scale [55], and the experiences with VA care, assessed by the Evaluation of VA Care scale, which is an 8-item scale measuring patient satisfaction with and perceptions of quality of VA healthcare [56]. We will also include 2 questions to assess general utilization of pain treatment in the past 6 months. At 3-months and 6-months, we will include a single item measure assessing how patients rate their quality of pain care at the VA in the past 6 months.

##### Pain treatment

In order to further assess use of opioid analgesics, we will obtain prescription information from electronic medical records. We will include survey questions to assess the use of opioids and non-opioid treatments for pain.

##### Sociodemographic and health-related factors

Participants will be asked to provide information about whether they use a walking aid and information (i.e., height and weight) used to calculate their Body Mass Index. We also will assess basic socio-demographic information such as education level, income, employment status, and disability status (e.g., worker’s compensation, Social Security Disability Insurance, VA service connection).

### Analysis plan

#### Power and sample size estimate

Our sample size calculation uses the RMD score as the primary outcome at six months. For our primary analysis we will use a responder analysis, in which we define clinically significant changes as a 30% reduction in pain disability from baseline, using the RMD, and have powered the study to be able to detect this change. This is the accepted threshold for clinically significant improvement in clinical trials and recommended by the IMMPACT guidelines [57, 58]. Previous studies demonstrate that a 30% reduction on the RMD is a clinically important difference [59, 60]. Prior studies have shown that 15-20% of UC patients demonstrate a 30% reduction in pain function score (using the RMD and similar measures) from baseline to follow-up [61, 62]. Thus, in order to detect an absolute difference of 20% in the primary outcome measure between treatment groups (i.e.15-20% responders in the UC group compared with at least 35-40% responders in the intervention group), we estimate that we need between 86 and 97 people in each group with complete data. This estimate takes into account that we set the alpha error rate to look at African American and non-African American patients separately [63, 64].

We factored in that up to 50% of the study sample might be non-African American. Since we want to have power to detect meaningful effects in the subgroup that only includes African American patients (~100/50% = 200), and to allow for up to 20% attrition, we will need to randomize 250 patients for each arm of the study, for a total of 500 patients recruited with at least 200 African American patients with complete data for the final analyses. For the purposes of this study, we are not forcing balance between African American and non-African American participants, and are instead allowing for the potential that more than half of the participants are African American, since assessing efficacy of the intervention in a population of African American pain patients is the primary target.

#### Primary aims

The primary aims are to test the hypothesis that the intervention will improve chronic pain-specific physical functioning—the primary outcome (H1a), improve emotional functioning, pain severity and ratings of overall improvement (H1b), and increase walking (H1c), compared to UC for African American patients with chronic hip, back, and knee pain.

After assessing data for normality and evidence of balance of baseline factors across intervention groups, generalized linear regression models will be used to estimate the main effects of the intervention for all participants (African Americans and non-African Americans).

To compare the effects of the intervention to UC on the IMMPACT measures of pain outcomes, the primary outcome measure is the RMD score assessed at 6 months (i.e. primarily a 30% reduction from baseline and secondarily mean change from baseline) (aim 1a). The secondary outcome measures are pain severity, the PHQ-8 and GAD-7, and the global rating of change scale (aim 1b). For all of these outcomes we will use generalized linear regression fit using appropriate distribution and link functions. Each participant will be modeled as a random intercept to allow within-patient correlation of the repeated measures. The use of a mixed-effects model will allow us to use data from participants who may be missing either baseline or the 6-month data while giving an unbiased estimate of the outcome comparisons as long as missing data is approximately random.

To compare the effects of the intervention to UC on increasing walking behavior (aim 1c), walking will be measured by step counts, which are reported based on readings from the Omron HJ-321 pedometer. The effect of the intervention on step counts will be assessed by comparing average daily step counts measured over 7 days. We will use a mixed-effects model with the average daily step counts during the last week of the three month and six month measurement points as the dependent variables, with a similar analytic plan as described for Aim 1a.

#### Secondary aims

Our secondary aim is to investigate whether key contributors to racial/ethnic disparities targeted by the intervention (motivation to exercise, pain/exercise efficacy, reduction of pain-related fear, increased physical activity) mediate improvement in chronic pain outcomes.

The key measures for this aim include the Fear-Avoidance Beliefs Questionnaire (FABQ) [51], the Exercise Regularly Scale [43], the Pain Self-Efficacy Questionnaire (PSEQ), and average daily step counts measured over 7 days [44]. Each of these will be measured at baseline, three and six month, and each has continuous distributions, and therefore, analytic approaches for each of these measures will be identical to that of Primary Aim 1 with the primary objective of assessing the long-term (6 month) effect of the intervention on these outcomes and secondary objective of exploring outcome trends over time. Indirect effects will be directly tested using the bootstrap approach to obtaining confidence intervals [65] to avoid the often-violated assumption underlying Sobel’s (1982) method that the sampling distribution of the indirect effect be normal [66].

Our secondary aim 2 is to explore whether the intervention reduces use of opioid analgesics. Using similar analytical methods as described for the Primary Aims, we will use generalized linear models to explore whether use of opioid analgesics are reduced in the intervention group compared to the UC group, using survey responses and pharmacy data. We will compare the proportion of participants who, at baseline, report taking opioids at baseline at the 6-month follow-up report that they no longer take opioids, using the question, “Do you take an opioid medication for pain? (Examples: codeine, Tylenol #3, hydrocodone, Vicodin®, hydromorphone, methadone, morphine, oxycodone, Percocet®, etc.).” We will also examine opioid daily dose reduction of 50% from baseline to 6 months, based on VA pharmacy dispensing data that has been converted to morphine equivalent doses.

Our secondary aim 3 is to determine if the intervention is effective for non-African American VA patients and other subgroups of patients who may experience barriers to effective pain treatment. We will also explore whether there are differences in effectiveness between African American and non-African American patients. We will conduct the same analyses as for the Primary Aims and Secondary Aims 1–2 on the sample of non-African American patients (most of whom are expected to be white) and subgroups based on key demographic factors (age, gender, education and income) and psychological, environmental and utilization factors, measured at the baseline survey that we included because they are expected to constitute barriers to effective pain treatment, based on our theoretical framework. We will also explore whether treatment effects will be moderated by common psychiatric conditions (anxiety and depression) and receipt of other forms of pain treatment.

### Limitations

UC patients will receive some intervention elements (e.g., they will receive a pedometer and some of the educational content). Nevertheless, we believe that the design represents the optimal compromise between scientific rigor and real-world practicality. Further, we expect that neither the pedometer nor the extra education will have a substantial influence on our primary outcome. If they do have any impact, the effect would be to reduce the likelihood of finding treatment differences between the control and intervention groups. Thus, the design for this study will provide rigorous evidence about the effectiveness of the proposed intervention.

Another limitation inherent to this design is that, if the program is effective, we will not be able to determine the specific intervention element responsible for the success of the program. We decided to test a multi-component intervention, rather than test different components of the intervention separately, in order to target key factors that we believe are necessary to increase walking in this population, and to ensure that the intervention was potent enough to affect change.

## Discussion

Presently, there is evidence of racial disparities in pain and pain treatment in the US but few interventions designed to specifically work well in populations most impacted by these disparities. This project adds to the evidence base on how to best improve pain treatment for African American patients with musculoskeletal pain, as well as other individuals who experience similar contributors to pain, and on the specific mechanisms that contribute to this reduction.

One innovative aspect of this study is that it uses proactive telephone outreach to increase physical activity among patients with musculoskeletal pain. In proactive telephone outreach, counselors reach out to patients, offering them treatment, as compared to “reactive care,” in which the individual patient must initiate treatment. Proactive outreach can address barriers that members of negatively stereotyped groups (such as minority patients and chronic pain patients) are likely to experience. For example, such patients may experience poor quality communication with providers, and discrimination within and outside the healthcare system, which is associated with avoiding and delaying healthcare (i.e., barriers to healthcare utilization) [1–3, 5–15, 32]. Our proposed intervention also applies action planning, which has been shown to increase the likelihood of behavioral change, to promote physical activity in patients with musculoskeletal pain, and is the only one that specifically focuses on minority pain patients. Action planning, which has been shown to be particularly effective for situations in which self-regulatory capacity is diminished [67], is likely to be helpful for African Americans, who experience many situations that diminish regulatory capacity, such as social exclusion, racial discrimination, and stereotype threat [67, 68]. Action Planning is also likely to be helpful for patients with pain, as pain diminishes self-regulatory capacity [69].

At the present time when we are in the midst of a public health crisis caused by increased prescription of opioids [70], with limited evidence about the lack of long term effectiveness for its use in treating of chronic musculoskeletal pain [71–73], it is particularly important that effective, nonpharmacological approaches to pain management be developed, and made widely available. Through a proactive outreach approach to delivering a nonpharmacological intervention, the present study has the promise of increasing both equity and quality of treatment for chronic musculoskeletal pain.

## Abbreviations

BMI: Body mass index
CBT: Cognitive behavioral therapy
CCDOR: Center for chronic disease outcomes research
FABQ: Fear-Avoidance beliefs questionnaire
GAD-7: Generalized anxiety disorder 7-item scale
IMMPACT: Initiative on methods, measurement, and pain assessment in clinical trials
IRB: Institutional review board
LISRES-A: Life stressors inventory
MI: Motivational interviewing
NIH: National institutes of health
PEG: Brief pain intensity and interference scale
PHQ-8: Personal health questionnaire depression scale without the suicidality item (7 items)
PSEQ-8: Pain Self-Efficacy questionnaire (8 items)
PTSD: Post-traumatic stress disorder
RE-AIM: Reach, effectiveness, adoption, implementation, and maintenance
RMD: Revised roland and morris disability questionnaire
UC: Usual care
US: United States
VA: Veterans affairs
VAMC: VA medical center
